# Developing digital competencies of nursing professionals in continuing education and training – a scoping review

**DOI:** 10.3389/fmed.2024.1358398

**Published:** 2024-06-13

**Authors:** Tim Tischendorf, Martina Hasseler, Tom Schaal, Sven-Nelson Ruppert, Maria Marchwacka, André Heitmann-Möller, Sandra Schaffrin

**Affiliations:** ^1^Faculty of Health and Healthcare Sciences, University of Applied Sciences Zwickau, Zwickau, Saxony, Germany; ^2^Faculty of Healthcare, Ostfalia University of Applied Sciences, Wolfsburg, Lower Saxony, Germany

**Keywords:** continuing education, further education, digital competencies, nursing professions, digital technologies

## Abstract

**Introduction:**

The German health and care system is transforming due to advancing digitalization. New technological applications in nursing, such as social and assistance robotics, artificial intelligence and legal framework conditions are increasingly focused in numerous research projects. However, the approaches to digitalization in nursing are very different. When integrating technologies such as robotics and artificial intelligence into nursing, it is particularly important to ensure that ethical and human aspects are taken into account. A structured classification of the development of digitalization in nursing care is currently hardly possible. In order to be able to adequately deal with this digital transformation, the acquisition of digital competences in nursing education programs is pivotal. These include the confident, critical and creative use of information and communication technologies in a private and professional context. This paper focuses on the question which specific training offers already exist at national and international level for nursing professions to acquire digital competences.

**Methods:**

A scoping review according to the PRISMA scheme was conducted in the PubMed and CINAHL databases. The search period for the scoping review extended from 2017 to 2024.

**Results:**

The selection of the studies took place by inclusion and exclusion criteria and the content-related orientation of the publications. After reviewing the titles and abstracts, eight studies were included. Of these, four were published in German-speaking countries and another four in international English-language journals.

**Discussion:**

The topic of digitization of the nursing professions and the question of how nurses can acquire digital competences is gaining international attention. Nevertheless, the research on explicit continuing education programs for nursing professions is still undifferentiated. No specific continuing education offer for the development of digital competences of nursing professionals was identified. Many authors remained at the meta-level when developing methodological concepts for the acquisition of digital competences. The systematic integration of digitalization into higher education and continuing vocational training is mentioned in the publications. The development of theory- and research-based educational frameworks, which can be used as a basis for curricula in nursing studies and continuing education, is highly recommendable.

## Introduction

Digitalization in the nursing context is a development that is neither being questioned nor stopped (1). New technologies in nursing are discussed in many publications (2–4). However, approaches to digitalization in nursing vary widely, digital tools operate at different levels, and a current structured classification of development and digitalization in nursing is hardly possible (2). Relatively many publications focus on robotics and robotic systems (4, 5). Research literature reports on the use of service and logistics robotics in nursing care contexts, social robotics, assistance robotics, mobilization robotics (2–5). Current literature discusses the potential of artificial intelligence in nursing, e.g., how it will change nursing (6). Artificial intelligence can probably improve and support the organization of patient processes and treatment plans and/or provide all relevant information that physicians and nurses need to make correct decisions and/or assist with repetitive or routine care tasks or medication management (7, 8). Another recent development appears to be “nurse chatbots” that can, e.g., assist in taking medications, improve adherence, or assist in the care and health of chronically ill people to ensure quick access to health information (9). Other digital developments are being explored in the context of information and communication technologies, which include projects such as telemedicine, telehealth, telenursing, computer-based documentation, or specific apps to support people with dementia (e.g., for cognitive stimulation) (1, 10, 11). Various publications also test digital monitoring and sensor technology for analyzing behavior or prevention of falls, pressure sores, or for measuring vital signs, etc. (12).

Digitalization in the nursing sector is ongoing and offers numerous benefits such as improved workflows and more efficient communication. However, nurses need digital competences to be able to use the new technologies effectively. The areas of nursing staff that would particularly benefit from digitalization include the organization of patient processes, the improvement of treatment plans and access to health information. It also improves patient care by enabling precise and individualized care and reducing the workload of nursing staff (1). Therefore, van Wynsberghe (13) sees the significance of competencies and expertise of nurses in the use of digital technologies to integrate the roles, relationships, responsibilities of all professional and personal groups involved in health care, as well as ethical and professional dilemmas. As a consequence, nursing professions need to acquire digital competencies in order to apply new digital tools appropriately in health care. This need exists not only in Germany, but also internationally, as digitalization is finding its way into healthcare worldwide.

International studies (14, 15) emphasize, among other things, technology acceptance by faculty in the educational context. The focus should be on both the use of technology in the classroom and the initiation of critical reflective competence in care practice. The acquisition of digital competences contributes to the professionalization of the profession, as emphasized by Sloane et al. (16). According to Ferrari (17) digital literacy includes: Knowledge, skills, and attitudes that enable to use information and communication technologies and digital media to accomplish tasks, solve problems, communicate, manage information, create and share content, and thus create a knowledge base and use it appropriately, effectively, creatively at the same time critically, autonomously, and ethically. Ferrari puts it this way:

“Digital Competence can be broadly defined as the confident, critical and creative use of ICT [information and communication technologies] to achieve goals related to work, employability, learning, leisure, inclusion and/or participation in society.” (17).

The aim of the scoping review is to investigate the extent to which ideally evaluated further and continuing education programs for the nursing professions in the field of digital skills already exist at national and international level and the extent to which the digitalization-related skills development of the nursing professions is discussed in nursing science. The primary research question aims to identify further education and training programs for the development of digital skills of nursing professionals, which ideally have already been evaluated. This has the potential to provide valuable insights into understanding the effectiveness of the programs and to identify or derive possible improvements. In addition, the article aims to provide an insight into the competence discourse around developments and trends in digitalization-related training in the nursing professions.

## Methodology

The Methodology of the systematic literature and database search was guided by the PRISMA scoping review process (18). The intent of the PRISMA-ScR is to help readers develop a greater understanding of relevant terminology, core concepts, and key items to report for scoping reviews. In order to provide as comprehensive an overview of the topic as possible, the following databases were initially used: PubMed from the National Library of Medicine and the Cumulative Index to Nursing and Allied Health Literature (CINAHL). Further hand searching was done in the journal database Educational Resources Information Center (ERIC), the subject information system Education (FIS Bildung) of the subject portal Pedagogy, in the database of funded projects of the German Research Association, Research Foundation (GEPRIS) and the Federal Ministry of Health in Germany. A supplementary search was performed via Google Scholar.

The database search was conducted in the period from March to April 2024. Different search strategies were implemented using appropriate search terms and Boolean operators. Thereby German terms and their English equivalents were applied, and terms were occasionally truncated:

(digitale Kompetenzen OR digital competence) AND (Pflege OR nursing) AND (Kompetenzentwicklung OR skills development) AND (Fortbildungsangebote OR further education offers) AND (Fortbildungen OR further education) AND (Technologieakzeptanz OR Technologie OR Akzeptanz OR technology acceptance) AND (Lehr-Lernumgebungen OR teaching-learning environment) AND (Lehr-Lernformen OR forms of learning OR forms of teaching) AND (Lehrende OR teachers OR instructors) AND (inhaltliches Wissen OR content knowledge) AND (berufliche digitale Kompetenz OR professional digital competences).

The following combination of search terms was used in CINAHL, as the programe’s user interface did not allow more than 12 search terms:

(digital competences) AND (nursing) AND (skills development) AND (further education) AND (technology acceptance) AND (teaching-learning environment) AND (teachers OR instructors) AND (content knowledge OR professional digital competence).

Studies from 2017 to 2024 were included and, according to the filtering capabilities of the databases, used flexibly to narrow down literature findings that were too extensive. The start of the search period in 2017 is based on the assumption that the research topics covered in the studies reflect the currently established use of digital technologies in nursing care. This also justifies the lead time required for the published studies and meta-studies. The filters used were:

- in PubMed: observational studies, reviews, systematic reviews, studies in the period 2017–2024
- in CINAHL: studies in the period 2017–2024

In the identification phase, 19.620 literature findings were organized in the PubMed and CINAHL databases and 931 literature findings were organized by hand searching additional sources using the Citavi 6 literature management and knowledge organization program. The hand search was based closely on the search terms of the database search. A total of 20.551 sources were searched for duplicates by the program and these were removed accordingly. The remaining number of publications was 20.066. In the pre-selection 152 publications were included. The pre-selection from the 20.066 publications was done by reading the headings according to the predefined inclusion and exclusion criteria. Included in the pre-selection were studies that – in addition to the reference to the research question – met the following criteria (Table 1).

**Table 1:** Inclusion and exclusion criteria for study preselection.

Inclusion criteria	Exclusion criteria
Published language: English or German language	Published language: No English or German language
Topics: Clear reference to digitalization-related continuing education Reference to nursing Reference to teachers at nursing colleges	Other topics: No reference to nursing or nursing science No relation to teachers at nursing colleges Focus on social media No reference to continuing education

**Figure 1:**
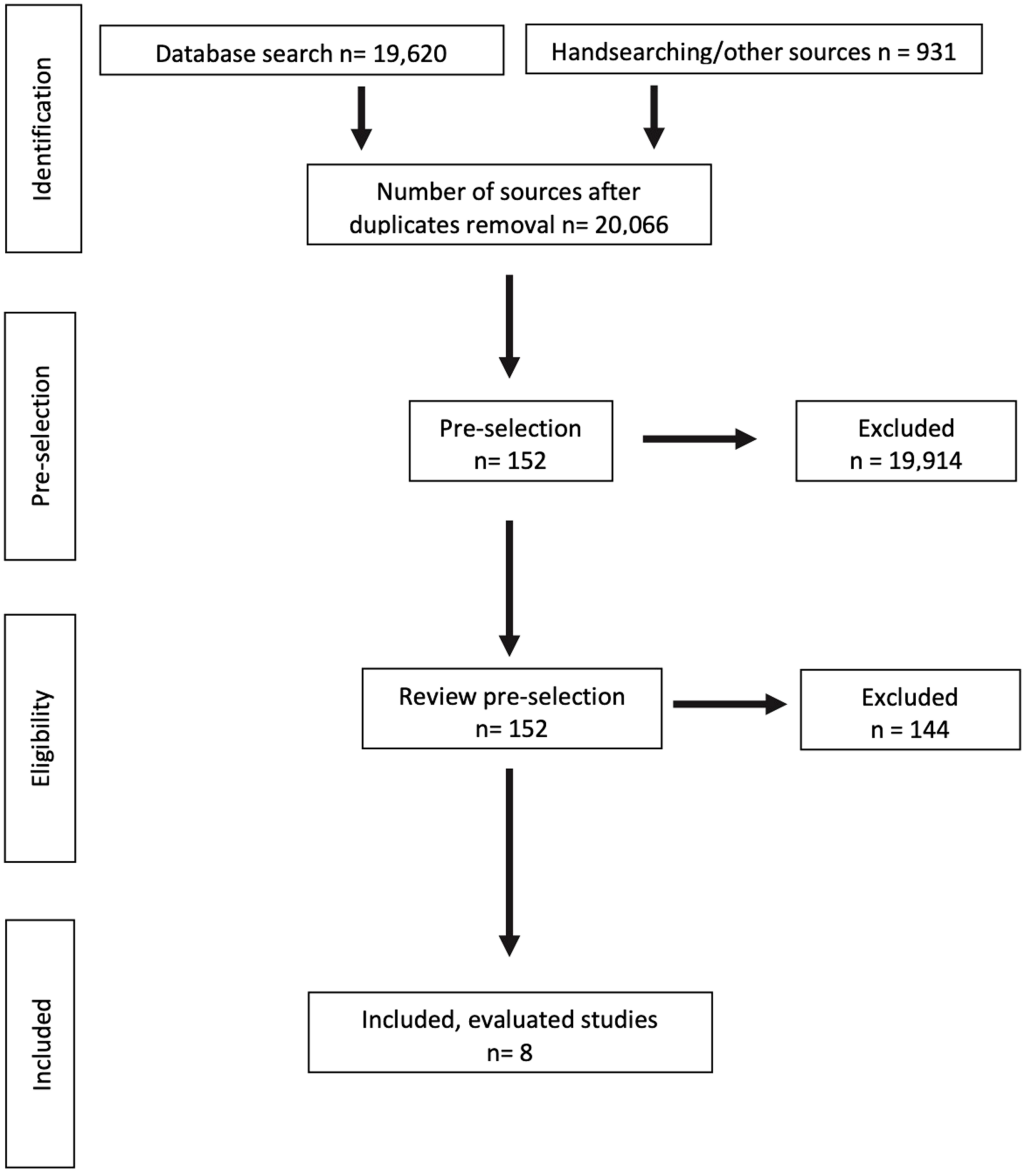
Flow chart of literature search and selection.

After the pre-selection was completed, the included texts were repeatedly checked for their suitability according to the research questions as well as the inclusion and exclusion criteria. This required downloading the full texts from the respective databases or publishers’ websites. In the first step, the abstracts were read, if available. If no abstract was available, a cursory reading of the full text was performed. In the second step, the extensive reading of the full texts took place. Of the 152 literature findings included in the pre-selection, eight sources were finally included in the analysis on the basis of the previously defined inclusion and exclusion criteria [cf., Figure 1 on the following page, based on Moher et al. (19)].

## Results

A total of eight studies were identified to address the research question (see Table 2). Of these, four publications were published in German-speaking countries and another four in international English-language journals.

**Table 2:** Overview of included literature.

Study no.	Country of origin	Study citation
1	Germany	Becka, D., Bräutigam, C., Evans, M., 2020. “Digitale Kompetenz” in der Pflege. Ergebnisse eines internationalen Literaturreviews und Herausforderungen beruflicher Bildung [“Digital Competence” in Nursing. Results of an International Literature Review and Challenges for Vocational Education]. Gelsenkirchen (Institut Arbeit und Technik. Forschung aktuell. 2020-08). https://hdl.handle.net/10419/224129.
2	Germany	Bleses, P., Busse, B., Friemer, A., Kludig, R., Breuer, J., Philippi, L. et al., 2018. Verbundprojekt KOLEGE – Interagieren, koordinieren und lernen: Chancen und Herausforderungen der Digitalisierung in der ambulanten Pflege [KOLEGE – Interacting, Coordinating and Learning: Opportunities and Challenges of Digitization in Outpatient Care]. Interim report – results of the analysis phase. 2nd, revised version. Bremen (Publication Series Institut Arbeit und Wirtschaft. 24). https://hdl.handle.net/10419/179718
3	Germany	Evans, M., Becka, D., 2021. Neue Herausforderungen für Personalentwicklung und berufliche Bildung in der Pflege [New challenges for personnel development and vocational training in nursing care], in: Friese, M. (Hrsg.), 2021. Care Work 4.0. Digitalisierung in der beruflichen und akademischen Bildung für personenbezogene Dienstleistungsberufe [Care Work 4.0. Digitization in vocational and academic education for person-related service professions]. Page 91–104, DOI: 10.3278/6004710w
4	Italy	Isidori, V., Diamanti, F., Gios, L., Malfatti, G., Perini, F., Nicolini, A. et al., 2022. Digital Technologies and the Role of Health Care Professionals: Scoping Review Exploring Nurses’ Skills in the Digital Era and in the Light of the COVID-19 Pandemic. In: JMIR nursing 5 (1), e37631. DOI: 10.2196/37631.
5	Finland/Japan	Konttila, J., Siira, H., Kyngäs, H., Lahtinen, M., Elo, S., Kääriäinen, M. et al., 2019. Healthcare professionals’ competence in digitalisation: A systematic review. In: Journal of clinical nursing 28 (5–6), Page 745–761. DOI: 10.1111/jocn.14710.
6	Singapore/Netherlands/UK	Nazeha, N., Pavagadhi, D., Kyaw, B. M., Car, J., Jimenez, G., Tudor Car, L., 2020. A Digitally Competent Health Workforce: Scoping Review of Educational Frameworks. In: Journal of medical Internet research 22 (11), e22706. DOI: 10.2196/22706.
7	Spain	Reixach, E., Andrés, E., Sallent R. J., Gea-Sánchez, M., Àvila López, A., Cruañas, B. et al., 2022. Measuring the Digital Skills of Catalan Health Care Professionals as a Key Step Toward a Strategic Training Plan: Digital Competence Test Validation Study. In: Journal of medical Internet research 24 (11), e38347. DOI: 10.2196/38347.
8	Germany	Seltrecht, A., Josupeit, F., 2021. Digital Media Educational Processes of Health and Nursing Professionals. Current Developments in Germany. In: Commission for International Adult Education. ED625445, Washington D.C.

The research literature identified on digital competence development in nursing can be divided into several clusters, each of which highlights different aspects of this topic. One cluster deals with the specific competence requirements of digitalization in nursing, examined by Becka et al. (20), Isidori et al. (21), and Konttila et al. (22). Another cluster deals with the implementation and evaluation of digital technologies in nursing, as examined by Bleses et al. (23) and Evans and Becka (24). Two further clusters are dedicated to the digital competence framework and level in healthcare, analyzed by Nazeha et al. (25) and Reixach et al. (26), and the integration of digitalization into education and training curricula, examined by Seltrecht and Josupeit (27) (Table 3).

**Table 3:** Overview of the findings in relation to the research question.

Study	Topic	Method	Findings in relation to the research question
Study 1 – Becka et al. (20)	Investigation of the empirically observed and scientifically discussed specific competence requirements of digitalization in nursing care	International literature review	The study on digital competence in nursing shows that English-speaking countries dominate the discourse and identifies three central areas of competence as well as the importance of ethical competences for all nursing staff. In addition, the implicit teaching of technical skills in nursing training curricula is pointed out and research desiderata for continuing professional development are formulated.
Study 2 – Bleses et al. (23)	Development, testing and evaluation of an implementation concept for the introduction of mobile digital assistants (MDA) in outpatient care, including a guideline for action	Practice-orientated action research (analysis, development, testing and evaluation)	It is pointed out that the potential of MDAs for formal and work-integrated learning in nursing has not yet been fully utilized. It is recommended that MDAs be integrated into an e-learning concept and that schools and practical assignments be linked, while further conceptual and content-related work in this direction is recommended.
Study 3 – Evans and Becka (24)	Investigation of the challenges of competence and personnel management resulting from the use of digital technology in interactive work processes in hospitals and nursing homes	Quantitative analysis (*n* = 1,214)	The study shows that a majority of respondents consider the benefits of digitalization in nursing care to be useful. At the same time, challenges such as technical disruptions and the fear of losing professional skills were mentioned. The results emphasize the need for a reflective approach and further research into the acquisition of digital competences in nursing, particularly in light of the redesign of the framework curricula for nursing training.
Study 4 – Isidori et al. (21)	Investigating the competences that nurses need in digital healthcare	Scoping review	Five main categories of digital competences for nurses were identified. The findings emphasize the need for specific training programs, the importance of a person-centered approach to communication, the positive role of nurses in telemedicine and the adaptability of experienced nurses to new technologies. It is called for these findings to be integrated into curricular training in order to do justice to future care pathways.
Study 5 – Konttila et al. (22)	Identification and description of key competencies for digitalization in the healthcare sector	Systematic review	The key competences identified in relation to digitalization from a healthcare perspective include knowledge of digital technology and the digital skills required for good patient care, including the associated social and communication skills, as well as ethical considerations for digitalization in patient care. Nurses must be motivated and willing to gain experience with digitalization in their professional context. The creation of a supportive working environment and collegial support are crucial to promote positive experiences with digitalization and to make the introduction of new technologies successful.
Study 6 – Nazeha et al. (25)	Investigating digital competence frameworks for healthcare professionals	Scoping review	The study identified a variety of digital health literacy frameworks relevant to nurses from higher income countries, targeting basic IT skills, management of health-related information, digital communication, and ethical and legal implications. Recommendations for future frameworks emphasize the need to consider emerging digital trends such as artificial intelligence and robotics and to include interprofessional competencies, taking into account low- and middle-income countries as well as medical students and non-physician health professionals.
Study 7 – Reixach et al. (26)	Identification of the digital competence level of Catalan healthcare professionals, as a basis for the development of a strategic training plan	Explorative observational study based on a survey	It was found that almost half of the respondents rated their digital competence at an intermediate level, with office tools being the most frequently used, followed by social media and electronic health documentation. Nurses mainly need training in the use of digital tools for health promotion, with self-assessment of their digital competence being lower compared to other professional groups, emphasizing the need for educational strategies for higher education.
Study 8 – Seltrecht and Josupeit (27)	Review of the extent to which curricula for initial and further training address digitalization in nursing and the extent to which the use of digital methods is included in the curricula.	Document analysis	The study shows that reflection on digitalization processes in society is not sufficiently represented in legal and curricular requirements for the nursing professions. It is emphasized that it is the responsibility of teachers to deal with digital media as learning objects and tools and their application in nursing care, whereby the development of a higher education didactic curriculum for the training of nursing teachers in this area is recommended.

## Study 1

Becka et al. (20) show in their study the state of research on digital competences in nursing, especially in an international context, where English-speaking countries dominate the discourse. They identify three main areas of competence: Core competences such as digital literacy and operating and application skills, specialized competences in data management and informatics, and reflexive competences such as ethical-reflective and social-communicative competences. In the literature, a narrowing of the discourse to competences in digitalization is noted and it is criticized that the allocation of competences to qualification levels and functional areas is problematic. The authors emphasize the importance of ethical competences for all nurses and have developed a scheme that combines digital and ethical competences. At the same time, they point to the implicit teaching of competences for the use of modern information and communication technologies in the framework curricula and curricula of generalist nursing training.

## Study 2

One of the interim results was the elaboration of the possibilities of formal as well as work-integrated learning with mobile digital assistants (MDA) (23). MDAs can be used as work-accompanying learning tools or in additional breaks as well as in home settings. MDA require use within the framework of an e-learning concept, in which various digital devices beyond MDA are included. They also see further potential here in the linking of nursing schools and practical assignments. They suggest conceptual and content-related work in this direction.

## Study 3

Evans and Becka (24) interpret the selection of results for an exploratory presentation of the connection between work, digitization and the appropriation of digitization in the professional field of nursing in hospitals. One aspect forms the “changes in the work process and participation”: here, 75% of the respondents rated the benefits of digitization in their workplace as useful in principle, 22% were involved in the development and 56.2% would participate more in the processes. The second aspect cited forms the “self-assessment, appropriation and competence challenges in the work process.” When dealing with technical failures, 30% of respondents said they did not know how to deal with them. At the same time, 46.1% agreed with the thesis that digital technologies are more error-prone. 80% of respondents stated that they would acquire the digital skills themselves. The second-to-last aspect cited was “correlations between digital competence, participation and discharge.” Here, 20% of respondents feared the loss of importance of their professional competencies. Those with a positive self-image of digital professional competencies (*n* = 199) also had a more positive evaluation than the other participants. The aspect “self-organization and potential consequences of a lack of real-time support” illustrates the connection between the digitalization of the company and the organization of work. At the same time, a retarding attitude of IT departments and hierarchical hospital structures was indicated. Evans and Becka (24) advocate the training of reflexive components. It is essential that nurses’ experiential knowledge and acquisition of competencies be further explored.

## Study 4

In their scoping review, (21) examined the skills of nurses in the context of the Digital Era and the COVID-19 pandemic. They distinguished five different categories from the included literature: communication skills in telemedicine, relationship management with patients in telemedicine, remote care of the chronically ill during the COVID-19 pandemic, management and leadership in the context of advanced telemedicine systems, impact of prior nursing professional experience on the use of telemedicine. Regarding the first category (communication skills in telemedicine), the need for specific training programs is confirmed, although a clear and effective training method remains undetermined. Traditional teaching methods are not considered appropriate for adequately teaching new technologies. Regarding the second category (relationship management with patients in telemedicine), communication in conjunction with a person-centered approach is highlighted as an important skill. At the same time, the work environment of nurses is important in this context so that uninterrupted communication can take place. The third category – remote care of the chronically ill during the COVID-19 pandemic – reflects positive experiences with “remote care” of ill people during the pandemic. Mainly, this concerned the educational aspect of telemedicine care and its flexible use, which helps overcome geographical and financial barriers. Regarding the fourth category (management and leadership in the context of advanced telemedicine systems), the authors elaborated on the increasing role of nurses as key players in the implementation of telemedicine in clinical practice. This requires specific leadership and management skills to set standards, develop plans, and overcome implementation barriers. In the last category (impact of prior nursing professional experience on the use of telemedicine), it was elaborated that professionally experienced nurses are better able to compensate for suboptimal functioning technologies than younger nurses due to their clinical practice experience. Also identified was the tendency of so-called digital natives to self-doubt when using digital tools. The first key finding cited by (21) is the structurally located leadership role of nurses in the course of introducing new technologies into the nursing process and service delivery. Another key finding is the need for the development of new communication, adaptation, and problem-solving skills. These skills are placed in relation to patients and their respective digital literacy. Finally, the introduction of cross-cutting, communication, and technology skills in the context of future care pathways into higher education curricula is called for.

## Study 5

In their systematic review, Konttila et al. (22) elaborated on various forms of digitization. These include telemedicine, telephone triage, telecare, electronic health records, wireless communication devices, medical technologies, computerized equipment, information-based patient education, health information technologies. The authors of the study divided the results into two categories: (a) competency area in digitization and related factors, and (b) the experience related to digitization. The competency domains named in the studies were conceptually defined as knowledge about telephone triage and telecare, skills in using health information technologies and attitudes toward these technologies, beliefs regarding the benefits or barriers of technologies, and lastly, motivation. Skill-related factors in the context of digitization were job position, workplace, team climate, and attitudes toward wireless communication devices. With regard to experiences concerning digitization, it was elaborated that digitization competence requires solid professional knowledge and skills. This includes recognizing ethical issues, autonomous decision-making, knowledge of clinical practice, and availability of a broad base of professional skills. Another aspect of professional competence related to digitization forms its influence by experience-based attitudes. In the literature reviewed, the negative attitudes of healthcare workers toward technology-related education formed a major finding. The third and final aspect identified by Konttila et al. (22) was the importance of psychological and organizational factors. These represent significant predictors with regard to the digitalization competence of employees in the healthcare sector. In the discussion, the authors emphasized the need for appropriate management and communication of digitization. In addition, healthcare workers must be given sufficient time and resources to adapt to new technologies. Learning how to use new devices should be integrated into everyday professional life. According to Konttila et al. (22), it is a leadership task for management to emphasize the benefits of the technologies to employees in the direction of improving daily clinical practice.

## Study 6

The majority of the included educational frameworks appeared to be useful for educational and practice-based application (25). They related to the specificity of the competencies, the organization of the competencies based on skill levels or professional roles, and the illustration of the competencies using setting-based case studies or practice examples (*ibid.*). Using the frameworks, Nazeha et al. (25) elaborated 28 competency domains: Administration and General Management, Analysis, Attitudes Toward IT, Clinical Service Delivery, Communication, Decision Support, Documentation, Education and Training, Ethics/Legal or Regulations, Financial Management, Health Information and Documentation Management, Quality of Care and Safety, Imaging, Informatics Concepts and Processes, Integration and Interoperability, IT Advocacy, Leadership and Management, Medication Management, Patient Access and Involvement, Security and Privacy, Project Management, Public Health, Remote Care (Telecare), Research, Risk Management, Systems Implementation, and Technical Knowledge and Support. The most frequently encountered domain was “informatics concepts and processes,” with another frequently represented domain in the frameworks being “health information and documentation management” and “communications.” At the same time, the domains “ethics/legal and regulations” and “security and privacy” had a significant importance. Domains from comparatively recent framework included “attitudes toward IT,” “medication management,” “IT advocacy,” and “public health.” In the authors’ perspective, these reflect trends in the digital skills that health professionals must possess. Other management-related domains indicate that health professionals need to consider organizational aspects in their use of digital technologies. The “communication” domain, in turn, also subsumes the social media aspect. In the overall view, Nazeha et al. (25) formulated recommendations for writing digital health literacy frameworks: The first aspect constitutes the method. They recommend conducting a literature review in combination with a Delphi study with local experts and later involving international experts. The second aspect is the content which should be explored even in existing frameworks (e.g., attitudes toward and advocacy for information technology (IT), medication management, public health). Another sub-issue here is updating competencies, building on technological innovations, their adoption, and consideration of emerging evidence (e.g., informatics concepts and processes, health information management, communication, ethics/law or regulations, security and privacy). Also interprofessional competencies should be included. The third aspect represents the target audience or setting to be included (nonphysician health professionals, low- or middle-income states). The fourth and final aspect of their recommendations focuses on application. Here, case studies or real-world examples should be integrated into the frameworks. Nazeha et al. (25) conclude that the majority of framework values target nurses and come from high-income states. They were created through an iterative approach. Existing frameworks focus on basic IT skills, skills and abilities in managing health-related information and digital communications, and awareness related to ethical, legal, privacy, and security implications. Future frameworks should consider the evolving nature of digitization and include emerging digital trends such as artificial intelligence and robotics. There is also a need for frameworks for low- and middle-income states, medical students, and other non-physician health professionals.

## Study 7

Nearly half of the respondents rated their digital literacy at an intermediate level (384; 47.8%); office tools were the most commonly used digital tools (750; 93.4%), followed by social media (700; 87.1%) and electronic health records (574; 71.5%) (26). In relation to the nursing profession, respondents mainly used office-based tools (206 of 227; 90.7%). In the mention of digital tools, social networks (198; 87.2%), electronic health documentation (192; 84.6%), telemedical tools (83; 36.6%), and digital health promotion tools (80; 35.2%) followed. The need for (additional) digital skills training was reflected by nurses as follows: In first place was training in the use of digital health promotion tools (106; 46.7%), followed by office tools (94; 41.4%), electronic health documentation (93; 41%), telemedicine tools (91; 40.1%), and lastly social networks (41; 18.1%). Regarding the test of digital literacy (up to 10 initial competencies, 10–25 basic competencies, >intermediate level of competency), the mean score was 22.6 (SD 4.3) of all respondents, with significant gender differences visible (m: 23.2; SD 4.2/w: 22.4; SD 4.3, *p* = 0.03). For nurses, the mean score of the competency test was 21.7 (SD 4.2). In light of the composition of the population from other health-related professional groups, it became clear that self-assessment of digital competencies was lower or not higher among nurses and medical professionals than among other professional groups (e.g., speech therapists had the highest self-assessment with a mean of 24.6 (SD 3.9)). Reixach et al. (26) illustrate that the need for training programs (continuing education programs) is widespread and that its characteristics were associated with the frequency of use. In light of this, the Catalan regional government will create an educational strategy for higher education training and continuing education for professional health care workers. According to Reixach et al. (26), the assessment and formal recognition of digital competences must be included in this process.

## Study 8

The degree of educational reflection on digitization processes in society is not found in the legal and curricular requirements (27). According to Seltrecht and Josupeit (27), it is up to the professional self-image of teachers to deal with digitalization as a process, i.e., with the examination of digital media as a learning object and tool as well as with the application of digital technologies in nursing. It is suggested that the topic of digitization be included in the qualification of teachers for nursing education in the form of a high-level didactic curriculum.

## Information on the risk of bias within the studies

The publication by Becka et al. (20) is a status report by the Institute of Labour and Technology. Limitations of the literature review are not mentioned. Bleses et al. (23) did not state any limitations. This is an interim report of a joint project that was still ongoing at the time. Evans and Becka (24) do not state any limitations, although they point out that the presentation of results is based on a selection from the overall results of their project (*ibid.*). Nazeha et al. (25) point out that they cannot claim to be exhaustive with regard to digital education-related framework concepts. At the same time, some of the identified frameworks are conceptually unclear, so that the classification of the frameworks led to discussions (*ibid.*). At the same time, the classification work by two reviewers does not rule out alternative approaches to interpreting the frameworks (*ibid.*). Isidori et al. (21) cite as limitations the fact that eHealth literacy was only analyzed in a few studies and due to different terminologies. Another limitation is the lack of concrete nursing experience with telemedicine approaches (*ibid.*). The lack of a European nursing curriculum as a basis for a strategy for the integration of technology-related skills is also seen as a limitation of the scoping review (*ibid.*). Konttila et al. (22) cite as limitations that the comparability of the studies is limited due to their validity and that the studies originate from countries with different socio-economic starting conditions. There were also considerable differences in digitalization in the respective countries from which the studies originated (*ibid.*). Reixach et al. (26) cite various limitations: Firstly, the test was not used in its original form as it was a practical test. There were also uncertainties with regard to response behavior and due to a selection bias, as more digitally competent people tended to take part in the survey (*ibid.*). No limitations were stated in the publication by Seltrecht and Josupeit (27).

## Discussion

In summary, it was not possible to identify any specific continuing education courses for the developments of digital competencies of nursing professionals in the literature. Probably the type of literature (scoping reviews, reviews, individual studies) could be a reason for this. Many authors remained at the meta-level in their comments.

The results of the scoping review are unassertive: Based on the current literature, the general impression is that specific and well-studied continuing education and training programs for the development of digital competencies of nursing professionals do not yet exist. Nevertheless, a development toward methodological concepts for external measurement of digital competencies as an alternative to self-assessment instruments (26), the regular integration of digitalization in higher education (21, 26), and continuing and further professional education [also (26)] is emerging. The situation in the German educational context remains unclear, as there are different interpretations of whether and to what extent digitalization is taken into account in educational contexts. This is discussed by various authors (20, 24, 27). Seltrecht and Josupeit (27) deny the mapping of this topic here, while Becka et al. (20) and Evans and Becka (24) assume an at least implicit consideration of this topic in the framework curricula. Becka et al. (20) attest a narrowness in the competence discourse to the English-speaking field, since in the literature originating from this area no social-communicative and reflective competences could be identified, which they consider relevant in the context of the development of digital competences.

Nazeha et al. (25) examine a variety of curricula or frameworks in their review and elaborate on a variety of domains for the development of digital competencies of nursing professions. Specific core domains that were frequently found in the curricula became visible. These reflect the approximate trends in digitization-related education in international nursing. At the same time, it becomes clear that in the international context – also against the background of COVID-19 – practical experiences can be drawn upon, which are analyzed accordingly in the scientific literature (21, 22, 26). Building on these experiences and the measurement of competencies [here (26)], the teaching of competencies is called for as a component of university curricula (21, 26), in continuing professional education and training (26), and in everyday professional life (22). The latter possibility is also addressed in the German context (23).

The literature included in this context illustrates the demand for overarching competence frameworks and the fundamental anchoring of the initiation of digital competences in nursing training. One possible research implication that can be derived from this is the development of teaching and learning modules that can be integrated into initial, further and continuing nursing training in order to instill digital competences in the nursing professions. In addition to the results reported here, further research work, such as on the sustainable integration of digitalization in the context of nursing training, can provide valuable insights into such module content (28). It should be noted that the targeted and efficient development of digital competences in nursing students can only be guaranteed if teachers at nursing schools already have a comprehensive level of competence in this area. With this in mind, a research project was launched at the beginning of 2023 in which the Federal Institute for Vocational Education and Training, the Ostfalia University of Applied Sciences and the West Saxon University of Applied Sciences Zwickau were commissioned to develop training and further education modules for teachers and practical instructors at nursing schools for the initiation of digital competences in the nursing professions (29). Only on the basis of this comprehensive perspective, which takes into account and includes all stakeholder groups involved in this transformation process, can digitalization in nursing be achieved.

In the context of this scoping review, it should be noted that the work does not claim to cover the topic in its entirety. This is explained on the one hand by the underlying research question, and on the other hand by the search operators and search filters. A further limitation arises from the literature itself, as it mainly provided meta-level insights. In this sense, this scoping review is already based on third-party interpretations. In order to transparently report the risk of bias within the included studies, a risk of bias analysis was conducted within the results section. Nevertheless, the work added value by preparing a thematic focus on specific continuing education programs for nursing professionals at the international and national level for the acquisition of digital competencies.

## Conclusion

The topic of digitization of the nursing professions and how nursing professionals can achieve digital competencies is gaining international attention. Nevertheless, international research on explicit continuing education programs for nursing professions is still undifferentiated. Furthermore, there seems to be a lack of original studies that investigate the development of digital competencies for nursing professions in a theory- and empirically-based manner. A promising perspective for the future is to develop theoretically based frameworks and modules that serve as the basis for curricula in degree programs and continuing professional development in order to optimally prepare nursing staff for the requirements of digital healthcare. The inclusion of digitalization and thus digital competences in nursing training should not be thought of and teached as an addition to existing training content, but should be understood as an integral part of nursing training in order to do justice to the digital transformation process.

## Author contributions

TT: Visualization, Writing – original draft, Writing – review & editing. MH: Conceptualization, Project administration, Writing – original draft. TS: Conceptualization, Writing – review & editing. S-NR: Writing – review & editing. MM: Conceptualization, Writing – review & editing. AH-M: Formal analysis, Investigation, Writing – review & editing. SS: Formal analysis, Investigation, Writing – review & editing.
